# Clinicopathological Correlations of Podoplanin (gp38) Expression in Rheumatoid Synovium and Its Potential Contribution to Fibroblast Platelet Crosstalk

**DOI:** 10.1371/journal.pone.0099607

**Published:** 2014-06-16

**Authors:** Manuel J. Del Rey, Regina Faré, Elena Izquierdo, Alicia Usategui, José L. Rodríguez-Fernández, Abel Suárez-Fueyo, Juan D. Cañete, José L. Pablos

**Affiliations:** 1 Servicio de Reumatología, Instituto de Investigación Hospital 12 de Octubre (i+12), Madrid, Spain; 2 Centro de Investigaciones Biológicas, Consejo Superior de Investigaciones Científicas, Madrid, Spain; 3 Unitat d’Artritis, Servei de Reumatologia, Hospital Clínic de Barcelona and Institut d’Investigacions Biomèdiques August Pí i Sunyer, Barcelona, Spain; University of Leuven, Belgium

## Abstract

**Introduction:**

Synovial fibroblasts (SF) undergo phenotypic changes in rheumatoid arthritis (RA) that contribute to inflammatory joint destruction. This study was undertaken to evaluate the clinical and functional significance of ectopic podoplanin (gp38) expression by RA SF.

**Methods:**

Expression of gp38 and its CLEC2 receptor was analyzed by immunohistochemistry in synovial arthroscopic biopsies from RA patients and normal and osteoarthritic controls. Correlation between gp38 expression and RA clinicopathological variables was analyzed. In patients rebiopsied after anti-TNF-α therapy, changes in gp38 expression were determined. Platelet-SF coculture and gp38 silencing in SF were used to analyze the functional contribution of gp38 to SF migratory and invasive properties, and to SF platelet crosstalk.

**Results:**

gp38 was abundantly but variably expressed in RA, and it was undetectable in normal synovial tissues. Among clinicopathologigal RA variables, significantly increased gp38 expression was only found in patients with lymphoid neogenesis (LN), and RF or ACPA autoantibodies. Cultured synovial but not dermal fibroblasts showed strong constitutive gp38 expression that was further induced by TNF-α. In RA patients, anti-TNF-α therapy significantly reduced synovial gp38 expression. In RA synovium, CLEC2 receptor expression was only observed in platelets. gp38 silencing in cultured SF did not modify their migratory and invasive properties but reduced the expression of IL-6 and IL-8 genes induced by SF-platelet interaction.

**Conclusions:**

In RA, synovial expression of gp38 is strongly associated to LN and it is reduced after anti-TNF-α therapy. Interaction between gp38 and CLEC2 platelet receptor is feasible in RA synovium *in vivo* and can specifically contribute to gene expression by SF.

## Introduction

Synovial fibroblasts (SF) are a heterogeneous cell population that represents the main resident cell component of synovial tissue. In rheumatoid arthritis (RA), SF expand and undergo phenotypic changes that contribute to the pathogenesis of chronic arthritis [1]–[3]. SF can respond to cytokines and, they maintain prolonged changes on the expression of genes involved in persistent inflammation and joint destruction in RA [4]–[6]. Crosstalk between SF and myeloid and lymphoid cell seems critical for persistent recruitment, survival and activation in chronic inflammation. These functions are associated to specific SF properties that resemble those of stromal cells in lymphoid tissues [7]–[10]. Lymphoid stromal cells play critical roles for the physiological trafficking and anatomico-functional compartmentalization of immune cells that supports normal immune responses [11], [12].

Among the shared lymphoid and RA stromal features, the expression of the surface glycoprotein podoplanin or gp38 has been reported [12]–[14]. gp38 expression is normally restricted to lymphatic endothelium and in lymphoid organs, to stromal cells of the T-cell zone. Aberrant expression of gp38 in fibroblasts has also been observed in other pathological tissues where fibroblasts play diverse roles in cancer progression or fibrosis [12], [15], [16]. gp38(+) fibroblasts might emerge in inflammatory tissues due to either specific cell proliferation of local gp38(+) progenitors or to induced expression in gp38(−) fibroblasts by inflammatory cytokines [14], [16], [17]. In a murine model of experimental autoimmune encephalomyelitis, a gp38 antagonist reduced inflammation-associated lymphoid neogenesis (LN) pointing to additional functions for gp38 in inflammation, although the precise mechanism remains unknown [18].

In cancer epithelial cells undergoing epithelial-mesenchymal transformation, gp38 expression confers enhanced cell migration and tumour invasiveness, consistently with the observation of gp38 up-regulation on the invasive front of tumors [19], [20]. In cultured lymphatic endothelium gp38 knockdown has also shown to reduce cell migration by regulating the activities of RhoA and Cdc42 GTPases [21]. This effect has been studied *ex vivo* and it seems mediated by indirect mechanisms of intracellular interaction between gp38 intracellular domains and ERM proteins ezrin and moesin that result in modification of small GTPase activities involved in cancer cell motility. Whether gp38 can modify cell motility in stromal cells of lymphoid organs or in inflammatory fibroblasts is not known.

The physiological and developmental functions of gp38 have been dissected in knockout mice. gp38 lacks intracellular signalling domains and its function seems to depend on its monogamous signalling receptor CLEC2. gp38 and CLEC2 knockout mice display an identical phenotype characterized by an embryonary defect in blood-lymphatic vascular separation [22]–[24]. In mice, CLEC2 is only expressed by platelets and some myeloid cell types, notably dendritic cells (DC) [25]. gp38 triggering of CLEC2 receptor induces platelet activation through Syk and SLP-76 signaling and this pathway seems critical for blood-lymphatic vessels partitioning during development [26], [27]. Crosstalk between lymphoid endothelial cells and platelets involves CLEC2 receptor triggering by gp38 and the release of specific platelet mediators that induce paracrine effects on endothelial cells [27].

To analyze the significance of increased gp38 expression in RA, we analyzed its correlation with clinical and pathological variables of the disease in a series of RA synovial tissues, including serial samples obtained before and after anti-TNF-α therapy. We also investigated the function of gp38 in SF by mean of RNA interference in different models of cell migration and platelet interaction relevant to RA pathogenesis.

## Material and Methods

### Patients and synovial biopsies

Synovial tissues were obtained from arthroscopic biopsies from the knee of patients who fulfilled the American Rheumatism Association revised criteria for RA (n = 38) [28]. All patients had active disease characterized by inflammation of at least one knee joint. Patients characteristics at biopsy were recorded and are shown in Table 1.

**Table 1:** Clinicopathological data of RA patients at biopsy.*

RA characteristics	
Age, years	60.6±12.5 [40–89]
Female	61.8%
RA duration, months	103.2±111.4 [4–441]
CD3+T cells/mm2	736±557 [9–2374 ] 2374 range]
CD20+B cells/mm	282±265 [2–1040]
CD68+cells/mm2	2267±1416 [45–1016]
DAS28	5.2±1.5 [2.34–8.35]
Lymphoid neogenesis positive (%)	63.9%
CRP (mg/dl)	3.5±2.8 [0.1–10.1]
Erosive disease	79.4%
RF positive^†^	61.2%
ACPA positive^†^	80.6%
Previous DMARD	100%
Previous anti-TNF-α	48.6%

*Quantitative data are expressed as mean±SD and [range]. CRP, C-reactive protein; DAS28, 28-joint Disease Activity Score; DMARD, disease-modifying antirheumatic drug; ^†^RF>30 IU/ml; anti-citrullinated protein antibodies ACPA>50 IU/ml.

Osteoarthritis (OA) synovial tissues were obtained by synovectomy at prostetic join replacement surgery (n = 15, age: 68±21, 60% female) and histologically normal synovial tissues were obtained from healthy individuals without joint disease at elective arthroscopy for minor traumatic lesions (n = 6, age 59±19, 60% female). In a subgroup of patients (n = 16), a second biopsy was obtained after at least 6 months of anti-TNF-α therapy. The study was approved by ethics committees of Hospital Clinic, Barcelona, and Hospital 12 de Octubre, Madrid, and a written informed consent was obtained from all patients.

### Immunolabeling of cells and synovial tissues

Immunohistochemical (IHC) staining was performed using a standard indirect avidin-biotin peroxidase method (ABC standard; Vector Laboratories, Burlingame, CA, USA) and developed by diaminobenzidine chromogen. The following antibodies and matched isotype controls were used: anti-gp38 mAb (D2/40 IgG1 clone, Dako, Glostrup, Denmark) [29], anti-CD61 mAb (SZ21 IgG1clone, Immunotech, Marseille, France), and anti-CLEC2 polyclonal goat Ab (R&D Systems, Minneapolis, MN, USA). Pretreatments included 10 µg/ml proteinase K (Sigma-Aldrich Quimica SA, Madrid, Spain) for CD61 detection, and microwave heating in 1 mM EDTA pH 8 for gp38 and CLEC2 detection. Double CLEC2 and CD61 immunofluorescent labeling was performed using sequential incubation with anti-goat IgG Alexa-Fluor 488 (green) and anti-mouse IgG_1_ Alexa-Fluor 594 (red) secondary antibodies (Invitrogen Molecular Probes, Eugene, OR, USA).

Synovial tissue sections were photographed and digitalized using a Spot RT CCD camera and Spot 4.0.4 software (Diagnostic Instruments, Sterling Heights, Michigan) on a Zeiss Axioplan-2 fluorescence microscope (Zeiss, Jena, Germany). gp38 immunoperoxidase stainned fractional area was quantified using ImageJ software (http://rsb.info.nih.gov/ij). IHC analysis and quantification of CD3, CD20 CD68, hsp47, CD31 in RA synovial biopsies as well as characterization of lymphoid neogenesis was performed as previously described [30], [31].

Fibroblasts grown on glass coverslips were immunolabeled with anti-gp38 mAb (D2/40 clone, Dako). Detection was performed with goat anti-mouse IgG_1_ Alexa-Fluor 594 labeled antibody and DAPI counterstaining. Photographs were obtained on a Zeiss LSM 510 META confocal microscopy (Zeiss).

Flow cytometric analysis of gp38 expression in cultured fibroblasts was performed with anti-gp38 mAb (clone 18H5, Santa Cruz Biotechnology Inc, Santa Cruz, CA, USA) [32] and goat anti-mouse IgG Alexa-Fluor 647 (Invitrogen Molecular Probes). Cells were analyzed on a BD FACSCalibur instrument (Becton Dickinson, San José, CA, USA).

### SF cultures and lentiviral siRNA transduction of fibroblasts

SF and normal dermal fibroblast (DF) cultures were established by explant growth of small biopsy fragments in Dulbecco's modified Eagle's medium (DMEM) supplemented with 10% heat inactivated fetal bovine serum (FBS) (Lonza, Verviers, Belgium) and used after 3^rd^ passage.

A small-interfering RNA (siRNA) targeting human gp38 and a scrambled non-silencing construction in pGIPz vector lentiviral were purchased from Open Biosystems (Thermo Fisher Scientific, Waltham, MA, USA). Lentiviral particles were obtained by co-transfection of 293T cells with pGIPz transfer vector, pPAX2 packaging vector, and pMD2-VSVg envelope vector as previously described [33]. Supernatants were harvested 48 hours after transfection, filtrated through a 0.45 µm filter and diluted 1/2 in DMEM medium for fibroblast transduction. Silencing efficiency was monitored by analyzing gp38 mRNA and protein expression by qRT-PCR and flow cytometry.

### SF migration and invasiveness studies

SF migration was analyzed in wound-healing assays. Cells were grown to confluence on culture chambered coverglasses (Lab-Tek II 4, NUNC, Rochester, NY, USA) and a wound was made by a single scrape using a sterile pipette tip. The wound area was sequentially photographed at 0, 18 and 24 h. The area covered by cells migrating into the wound area was determined using ImageJ software as the average closed area of the wound at 18 and 24 h after scraping.

The invasive capability of RA SF was analyzed using a matrigel coated transwell system (BioCoat Growth Factor Reduced Matrigel Invasion Chamber, Becton Dickinson). SF were resuspended in DMEM 0.1% BSA, plated on the upper chamber of the transwell insert (8 µm pores) and allowed to migrate through the matrigel matrix membrane for 4 days. DMEM with 10% FBS and 10% human serum was added to the bottom well and used as chemoattractant. The membrane was fixed with 4% paraformaldehyde and stained with DAPI for direct fluorescent microscopy. Ten random fields per membrane were photographed and digitalized and the number of cells per area was counted using ImageJ software.

The capacity of SF to invade cartilage was indirectly determined as the release of glycosaminoglycan (GAG) degradation products from cartilage SF cocultures. Fresh frozen human cartilage slices of fixed diameter and height (6×2 mm) were collected from cartilage obtained during joint replacement surgery for hip fracture and attached to 24-well plates. SF were dropwise added on top of the slices. After incubation for 3 h at 37°C and 5% CO_2_, wells were filled with 1 ml DMEM 10% FBS. Supernatants were removed after 4 and 7 days of culture and GAG determined using Blyscan assay (Biocolor Ltd, Belfast, N. Ireland) according to the manufacturer's protocols.

### SF platelet co-cultures

Platelets were isolated from peripheral whole blood in EDTA from healthy donors. Platelet-rich plasma (PRP) was obtained by centrifugation at 150 g at room temperature for 10 minutes. Platelets were pelleted, resuspended on DMEM and added to SF cultures at 1∶1000 ratio (SF:Platelets). SF were seeded into 6-well plates in 1% FBS DMEM one day before platelet co-culture. Co-cultures were maintained at 37°C for 5 h. Cells and supernatants were collected for RNA and protein quantification. Parallel SF cultures without platelets were established and used as controls.

Quantitative Real Time-PCR (qRT-PCR) of RNA extracts from SF was carried out on Applied Biosystems 7500 Fast Real-Time PCR System using either Power Sybr Green PCR Master Mix or TaqMan Gene Expression assays (Applied Biosystems). The sequences of PCR primers used are listed in Table S1. For relative quantification, we calculated the amount of target gene normalized to the endogenous reference gene (β-actin) using 2^-ΔΔCt^ formula, where Ct is the mean of threshold cycle at which the amplification of the PCR product is first detected. We performed a previous validation experiment comparing the standard curve of the reference and the target to demonstrate that the primers efficiencies were approximately equal.

IL-6 concentration in supernatants from SF platelets co-cultures was determined by ELISA (Biolegend Inc, San Diego, CA, USA).

### Statistical Analysis

Data were analyzed using Prism software v5.0 (GraphPad Software, San Diego, CA, USA). Results are expressed as mean±SD and means were compared by Student's *t*-test, Mann-Whitney U-test, and Wilcoxon tests as appropriate. Correlation between different numerical variables was analyzed by Spearman's or Pearson's tests as appropriate. Categorical variables were analyzed by Chi-square test. In all experiments, p value <0.05 was considered statistically significant.

## Results

### Clinicopathological correlates of gp38 expression in RA synovial tissues

**Figure 1:**
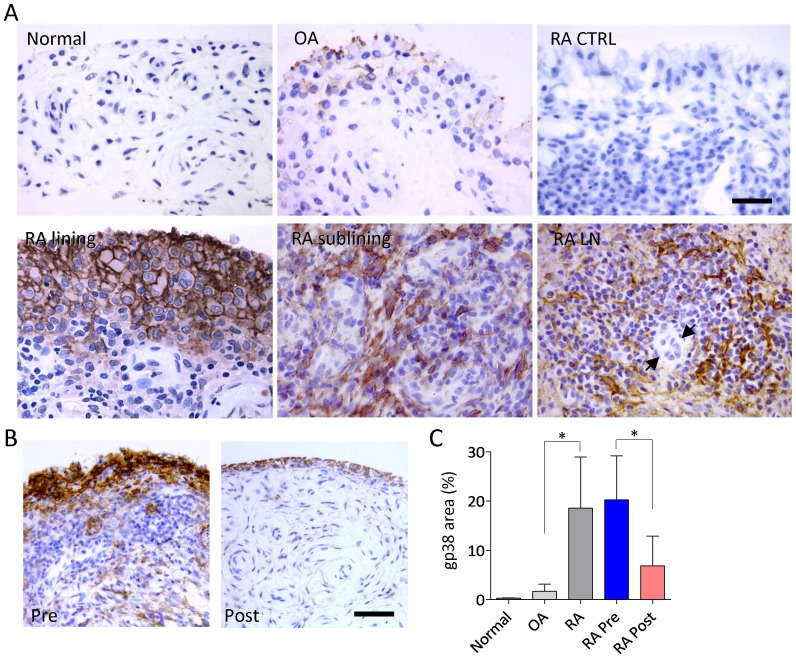
Expression of gp38 in synovial tissues. **(A)** IHC immunoperoxidase labelling of gp38 expression in normal, OA and RA tissues. RA CTRL: IgG1 isotype control. Lining and sublining areas, and a large lymphoid aggregate centered by a HEV (arrows) are shown. Bar: 50 µm. Original magnification 400x **(B)** Changes in gp38 expression after anti-TNF therapy. Representative imunoperoxidase labeling of gp38 of a single patient pre- and post-treatment. Bar: 100 µm. Original magnification 200x **(C)** Mean±SD gp38 immunostained fractional area in the different groups (OA vs RA Mann Whitney test *p<0.0001), and in basal (Pre) and post-anti-TNF-α therapy (Post) in 16 patients (RA Pre vs RA Post Wilcoxon matched-pairs signed rank test *p<0.0001).

To extend previous observations by Ekwall *et al* in advanced RA disease undergoing joint replacement [14], to RA patients biopsied at earlier stages, we performed quantitative IHC analyses in arthroscopic biopsies from a series of RA patients with active arthritis of the knee, heterogeneous regarding other characteristics of the disease (Table 1). Abundant gp38 expression was observed in synovial lining and sublining fibroblasts in RA biopsies (n = 38), whereas minimal expression was observed in OA (n = 15), and it was undetectable in normal synovial tissues (n = 6). In all RA tissues, gp38 was observed in lining cells, whereas in 54% of the cases it extended to sublining fibroblasts, and stromal cells within LN structures (Figure 1A). Lymphatic gp38(+) vessels were extremely rare in the superficial lining and sublining areas used for quantitative evaluation, but were readily observed in deeper tissues.

The level of gp38 expression in RA, expressed as the fractional labeled area, did not correlate with age, disease duration, activity evaluated as DAS28, C-reactive protein, macrophagic, T-cell or B-cell infiltration, and fibroblast or vascular density (data not shown). gp38 expression was found significantly increased in the group of patients (64%) with ectopic LN (Table 2). We also found significantly increased gp38 expression in the groups of patients with rheumatoid factor or anti-citrullinated protein (ACPA) autoantibodies compared to seronegative patients (Table 2). No correlation between LN and the presence of autoantibodies was found.

**Table 2:** Clinicopathological correlations of gp38 expression* in RA synovial tissue.

RA variables	gp38 expression*	p value
RF(+/−)	20.1±10.6/13.0±7.9	0.0078
ACPA(+/−)	20.4±10.2/12.3±7.9	0.0450
LN(+/−)	21.9±10.4/10.6±5.6	0.0004
Erosive disease(+/−)	18.9±10.6/15.0±8.0	0.22
Earlier disease^¶^(+/−)	19.5±7.4/18.2±10.8	0.55

*Fractional immunolabeled gp38 area (%, mean±SD). RF, rheumatoid factor; ACPA; Anti-citrullinated protein antibodies; LN, lymphoid neogenesis. ¶Patients with RA duration less than 1 year. Mean±SD.

In a subgroup of 16 patients that initiated therapy with a TNF-α antagonist after biopsy, we analyzed the changes in gp38 expression after therapy. In synovial tissues obtained after 12.3±14.1 months of anti-TNF-α therapy, a significant reduction in gp38 expression compared to pretreatment tissues was observed (20.2±9.0% to 6.9±6.0%, p = 0.0001) (Figure 1B). EULAR good or moderate responses [34] had been achieved in 12 of 16 patients at second biopsy. A lower decrease on gp38 expression in the second biopsy was observed in non-responders but the difference was not statistically significant.

### gp38 expression and function in cultured fibroblasts

**Figure 2:**
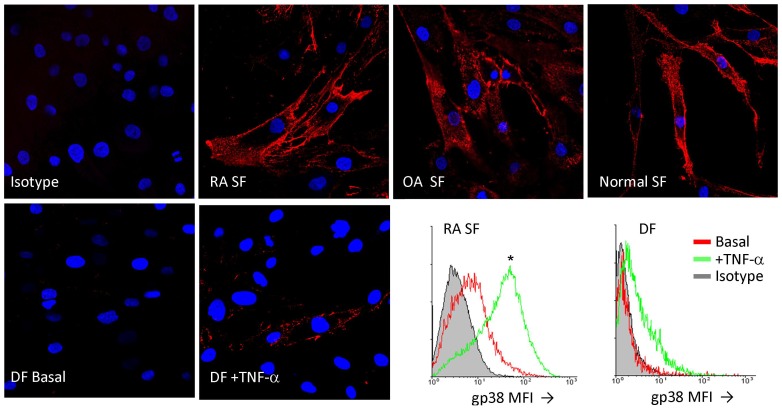
gp38 expression of cultured synovial fibroblasts. SF from RA, OA and normal synovial tissues, and normal skin dermal fibroblasts (DF) were cultured on glass coverslips and immunolabeled (red) for gp38 expression. TNF-α treated DF cultures are also shown (DAPI nuclear counterstaining). Flow cytometric detection of surface gp38 by in RA SF and DF untreated (basal) or treated with TNF-α for 24h. Data are representative of 6 SF and 3 DF lines (*p = 0.03 basal vs TNF-α treated). MFI: Mean fluorescence intensity.

Cultured SF displayed abundant gp38 expression irrespective of their source (RA, OA or healthy synovium) but this was not a general feature of cultured human fibroblasts since DF did not show gp38 expression (Figure 2). Membrane expression of gp38 was also confirmed by labeling of unpermeabilized SF by flow cytometry and confocal microscopy (Figure 2). Treatment of cultured SF with TNF-α induced a significant up-regulation of gp38 mRNA and protein expression, as determined by qRT-PCR (data not shown) and flow cytometry (Figure 2). In cultured DF, TNF-α also induced gp38 mRNA and protein expression but to a lower extent compared to SF.

To analyze the potential role of gp38 on the cell behaviour of SF, we performed RNA interference experiments in cultured RA fibroblasts. In SF transduced with a lentiviral gp38-specific siRNA construct, a profound downregulation of gp38 mRNA and membrane protein was observed compared to control siRNA transduced SF (Figure S1A). Cell viability was similar in gp38 (82.51±15.29%) and control (84.09±11.88%) transduced cells.

Since the best described function of gp38 is a gain of function in cell migratory and invasive properties of epithelial cancer cells [19], [20], we compared cell migration and invasive capacities of SF transduced with gp38 or control siRNA lentivirus. Cell migration through matrigel coated transwells was similar in gp38 silenced and control SF (Figure S1B). Invasion of wound area on plastic monolayer was also similar in gp38 silenced or control SF (Figure S1C), therefore excluding a relevant function for gp38 in cell adhesion and motility on matrigel or plastic surfaces in SF.

Previous studies have shown that RA SF attach and invade the cartilage causing degradation of the extracellular matrix. Therefore, to analyze the invasive capacity of SF in a model relevant to RA, SF were seed onto human cartilage slices [35], [36]. Transduced RA SF expressing GFP and siRNA efficiently attached and invaded cartilage *ex vivo*, as observed by direct fluorescent microscopy, and induced an increase on GAG release to the media. GAG release at 4 and 7 days after SF seeding was similar in gp38 silenced compared to control RA SF, excluding an important role for gp38 membrane expression in this process (Figure S1D).

### CLEC2 expression in RA synovial tissues

**Figure 3:**
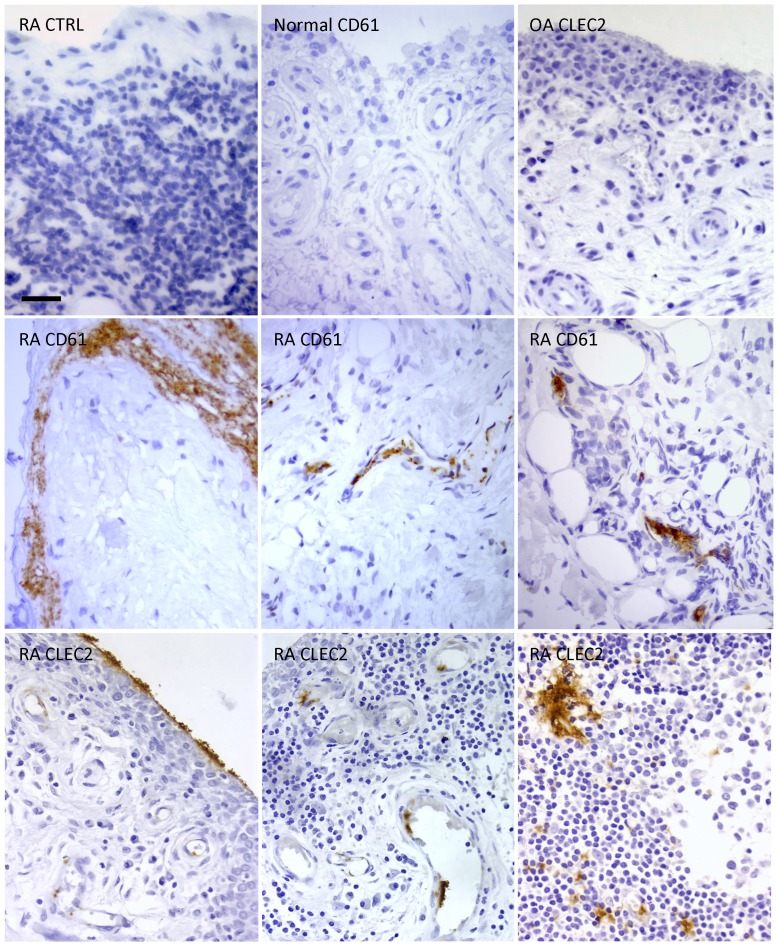
Detection of CLEC2 expression in synovial tissues. IHC immunoperoxidase labelling of CLEC2 receptor and CD61 (platelets) in OA, RA and normal synovial tissues as indicated. Non immune serum control for anti-CLEC2 IHC is also shown (RA CTRL). Bar: 50 µm. Original magnification 400x.

To identify expression of CLEC2 receptor in cells potentially interacting with gp38(+)SF, we performed IHC studies in RA synovial tissues. CLEC2 expression was only detected in small particles or aggregates lacking nuclei located in intra- and perivascular areas of the sublining area. In some cases, CLEC2 positive aggregates were associated to amorphous fibrin deposits attached to the lining surface. In these locations we demonstrated the presence of platelets by specific CD61 IHC (Figure 3). Double immunofluorescent labeling confirmed colocalization of CLEC2 and CD61(+) platelets (Figure S2).

Although IHC observations did not support CLEC2 expression by myeloid cells, to exclude lower levels of gp38 signalling receptor CLEC2 expression in human myeloid DC as recently described in murine DC [21], we performed western blot analysis of platelet and human DC under different maturation and activation status. Our data confirmed CLEC2 expression in platelet extracts but failed to detect CLEC2 in human DC (Figure S3). By flow cytometry, we also failed to detect binding of gp38-Fc protein to mature or immature DC (data not shown).

### gp38 function in fibroblast CLEC2 platelet interaction

**Figure 4:**
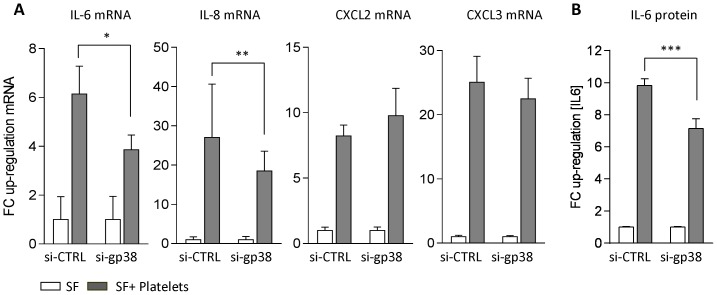
Expression of cytokines and chemokines in gp38 silenced RA SF platelet co-cultures. **(A)** Magnitude of the up-regulation on IL-6, IL-8, CXCL2 and CXCL3 mRNA expression induced by platelets and expressed as the ratio (FC) between SF and platelet co-cultures compared to parallel SF only cultures in gp38 siRNA silenced SF and non-silenced controls (si-CTRL) as measured by qRT-PCR. **(B)** IL-6 protein levels in supernatants of platelet cocultured gp38 siRNA silenced SF compared to non-silenced controls as measured by ELISA. Mean±SD data are representative of 3 independent experiments (*p = 0.0002, **p = 0.01, ***p<0.0001).

Since CLEC2 was not detected in any other cellular element of RA synovium, we focused on platelet SF functional interactions to analyze the potential function of gp38 in this system. Gene expression of cytokines, chemokines and metalloproteinases factors previously associated to proinflammatory or tissue destructive functions of RA SF was analyzed in SF platelet co-cultures compared to parallel SF cultures in the absence of platelets. Platelet co-culture induced a strong increase in the expression of IL-6 and the chemokines IL-8, CXCL2 and CXCL3 mRNA (Figure 4A), whereas MMP (MMP-1, MMP-3 and MMP-9 mRNA) and LN stroma related factors were non-significantly increased (IL-7, CXCL13 mRNA) or decreased (CCL21 mRNA) (data not shown).

In platelet cocultured SF, gp38 silencing significantly reduced IL-6 and IL-8 mRNA expression, whereas CXCL2 and CXCL3 mRNA expression was not significantly modified (Figure 4A). Protein levels of IL-6 in the supernatant of SF platelet co-cultures were also significantly reduced in gp38 silenced SF compared to non-silenced controls (Figure 4B).

## Discussion

Our data confirm that fibroblast gp38 expression is characteristic of RA inflammatory tissue, in contrast to normal synovial tissue, where it is undetectable. Interestingly, regardless *in vivo* expression, both normal and arthritic SF acquire similar membrane gp38 expression in culture. Therefore, exposure to growth factors and tissue culture conditions seem to induce gp38 in normal SF that do not express the protein *in vivo* but not in normal DF pointing to different tissue-specific fibroblast phenotypes. TNF-α and inflammatory cytokines may also induce gp38 expression in SF as previously reported [14] but again at much higher levels in SF compared to DF. Therefore, the higher capacity of SF to express gp38 represents a tissue specific property potentially relevant to their participation in joint specific inflammatory diseases. Recent studies have identified the proliferation of specific perivascular stromal precursors characterized by constitutive expression of gp38 and ADAM12 as the source of gp38 fibroblasts in different inflammatory mouse models [16], [17]. We failed to detect perivascular gp38(+) cells in human healthy skin (data not shown) and normal synovium. This observation, together with the uniform acquisition of gp38 expression by cultured SF and its induction by TNF-α, suggest that growth factor or cytokine induced expression may underlie the observed gp38 stromal expansion in RA synovium. In addition, gp38 was significantly downregulated in patients treated with TNF-α antagonists further supporting this mechanism.

The expression of gp38 in RA tissues from patients with advanced destructive disease had suggested its potential role in structural damage [14]. We have also identified high gp38 expression in patients at earlier phases of the disease, excluding a correlation between disease progression and the level of gp38 expression and indicating that gp38 expression may be an early feature in RA. No significant correlations between the level of gp38 expression and most clinical or pathological RA features were found, with the striking exceptions of LN and RA autoantibodies positive groups. The finding of increased gp38 expression in patients with synovial LN, together with the previous observation of defective lymphoid development in gp38 deficient mice, point to a mechanistic link between both processes [18]. In several mouse models of inflammation also characterized by LN, the expansion of gp38(+) fibroblasts has been demonstrated [17]. The dependence of inflammatory LN on gp38 has been demonstrated in the mouse model of autoimmune encephalomyelitis, where anti-gp38 antibodies abrogated LN development [18]. However, the role of gp38(+)stromal cells is uncertain in that study, since gp38 expression and function was attributed to Th17 cells.

Whether ontogenic or pathological LN is dependent on functional gp38/CLEC2 receptor interaction is unknown. Such interaction is feasible in RA, where we observed both gp38(+) SF and CLEC2(+) platelets in the synovial tissue. Previous studies had also demonstrated the presence of abundant platelet microparticles in the synovial fluid of patients with RA ant their capacity to induce potent paracrine proinflammatory effects on SF [37], [38]. Platelets respond to CLEC2 triggering by phosphorylating its cytoplasmic immunoreceptor tyrosine-based activation motifs (ITAM) which, in turn, recruits and activates tyrosin kinases Syk/Btk resulting in platelet activation. This process leads to the generation of platelet microparticles and the release of mediators stored in platelet granules such as IL-1 with potent paracrine effects on SF [27], [38]. ITAM-dependent platelet activation may also be induced by GPVI receptor and therefore, both pathways might participate in platelet-stromal inflammatory crosstalk. CLEC2 specific effects, not triggered by GPVI receptor activation, have been proposed using CLEC2 deficient platelets but are difficult to dissect out in inflammatory models where platelet activation is required to maintain vascular integrity [27], [39].

Our data confirm that platelet-SF co-culture induces strong changes in the expression of multiple genes by SF, similar to those induced by platelet microparticles [37], [38]. Among these genes, IL-6 and IL-8 were specifically reduced by gp38 silencing in SF in contrast with other platelet-induced genes, providing evidence of a contribution of CLEC2 to proinflammatory platelet-SF crosstalk. IL-6 plays a pivotal role in RA pathogenesis and its therapeutic targeting is highly effective in modifying the course of the disease [40]. Although macrophagic cytokines IL-1β and TNF-α are also potent stimuli for IL-6 expression in RA SF, our and previous data suggest that platelets may also play an important role [37]. However, the link between this observation and the potential role of gp38 expression in rheumatoid LN is unclear. IL-6 has pleiotropic functions on lymphoid cells including the development of Th17 cells as well as T follicular helper and B cells involved in the development of orthotopic germinal centres in autoimmune arthritis but its influence on ectopic LN development is unknown [41]-[43]. We did not detect gp38 dependent changes on the expression of stromal factors associated to ontogenic or inflammatory LN such as IL-7 or lymphoid homing chemokines CXCL13 or CCL21 [10], [44].

We failed to confirm an intrinsic role for gp38 in an *ex vivo* model of SF invasion and cartilage degradation, in contrast to that observed in cancer cells [19], [20]. In cancer epithelial cells, gp38 is associated to EMT, a process that provides mesenchymal cell migratory properties to epithelial cells otherwise lacking motile or invasive capacity. It is therefore possible that gp38 does not provide these functionalities to fibroblasts which are highly mobile cells. An alternative explanation is that siRNA silencing of gp38 expression is not complete and remaining gp38 expression might be sufficient to maintain its function.

In conclusion, our study shows a new mechanism involved in platelet-SF proinflammatory interactions in RA synovium, supporting an important role for the acquisition of gp38 expression in SF. We also identify a strong association between gp38 expression and synovial LN, consistent with its reported role in developmental and experimental LN, but the mechanistic basis of this association requires further studies.

## Supporting Information

Figure S1 **Migratory and invasive capabilities of gp38 silenced RA SF.** **(A)** Silencing efficiency of siRNA lentiviral transduction of RA SF as analyzed by qRT-PCR (si-CTRL mRNA gp38/β-actin ratio set to 100%) and flow cytometry (percentage of gp38(+) RA SF is indicated, *p =  0.03). MFI: Mean fluorescence intensity. **(B)** Invasive capability of RA SF on matrigel coated transwells expressed as the number of cells per field that migrated through the matrigel at 4 days. **(C)** SF migration in wound assays expressed as the percentual closure of the wound area 18 h and 24 h after scraping. A representative image is shown (100x). **(D)** GAG release into the supernatant at 4 and 7 days of RA SF cartilage co-culture. Basal: GAG release by cartilage in the absence of SF. A representative image of siRNA GFP transduced SF attached to cartilage is shown (200x). Results are representative of three independent experiments (NS: not significant).
(TIF)

Figure S2 **Double CLEC2 and CD61 platelet labeling in RA synovial tissues.** Double green (CLEC2) red (CD61) labeling with DAPI counterstaining is shown is shown in upper row as indicated. Controls (CTRL) including only anti-CLEC or only CD61 primary antibody and both secondary antibodies are shown in lower rows as indicated. Bar 20 µm.
(TIF)

Figure S3 **Western blot analysis of CLEC2 expression.** Platelets were obtained from healthy donors peripheral blood. Immature DC (imDC) were derived from buffy coat monocytes cultured for 7 days in complete RPMI medium containing GM-CSF (1000 U/ml) and IL-4 (1000 U/ml). For maduration, imDC were treated with LPS (1 µg/ml) or TNF-α (50 ng/ml) for 24 or 72 h respectively. Protein extracts (30 µg) are analyzed by western blotting with anti-CLEC2 polyclonal antibody (R&D Systems) or anti-β-actin mAb (Sigma), developed by peroxidase-conjugated secondary antibodies and ECL system.
(TIF)

Table S1 **Primer sequences used for quantitative real-time PCR analysis.**
(DOC)
