# Supplementary figures and images for: Clinicopathological Correlations of Podoplanin (gp38) Expression in Rheumatoid Synovium and Its Potential Contribution to Fibroblast Platelet Crosstalk
Source: PLoS One. 2014 Jun 16;9(6):e99607. doi: 10.1371/journal.pone.0099607 (PMC4059710; doi:10.1371/journal.pone.0099607)

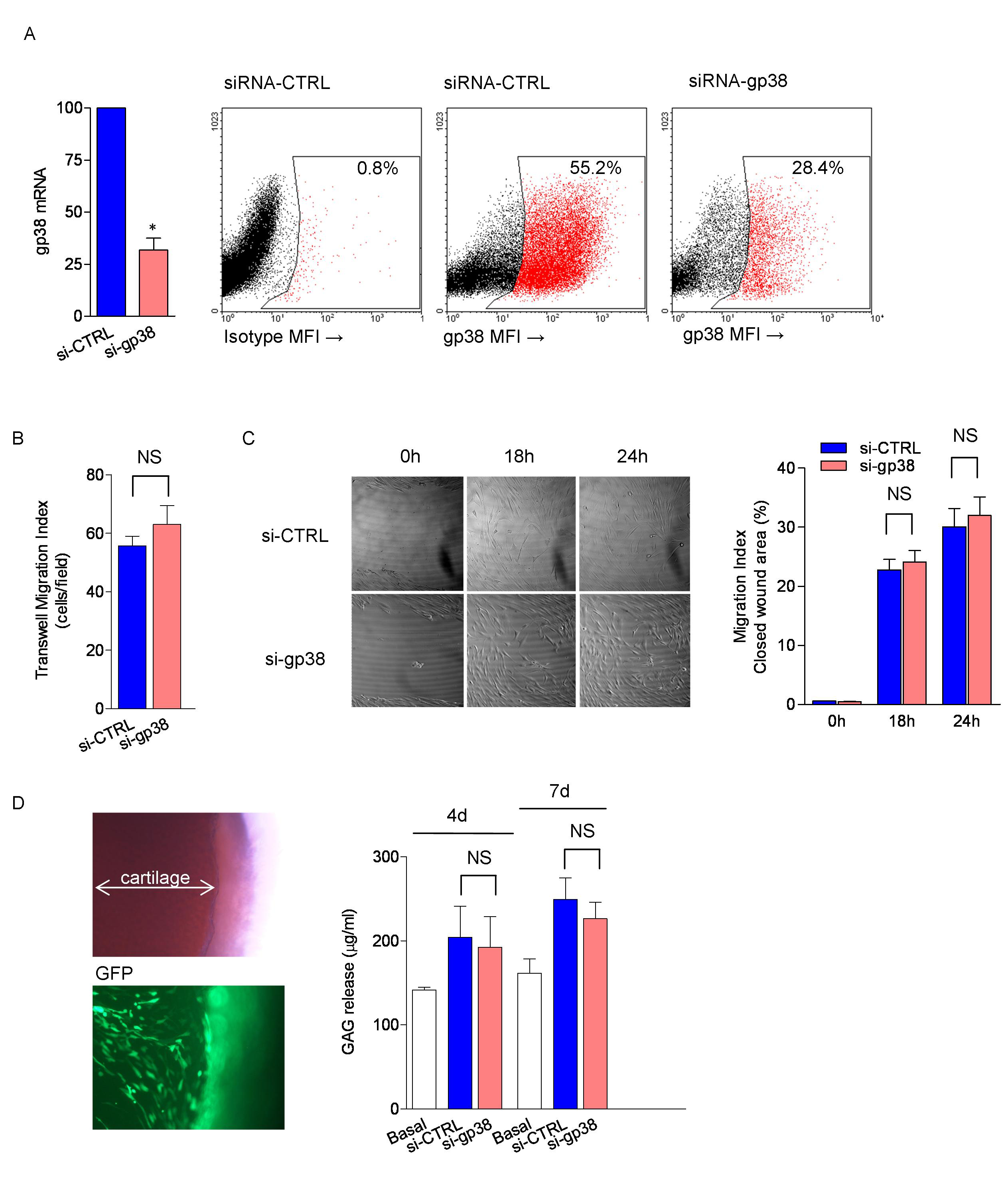

Supplement: Figure S1 — Migratory and invasive capabilities of gp38 silenced RA SF. (A) Silencing efficiency of siRNA lentiviral transduction of RA SF as analyzed by qRT-PCR (si-CTRL mRNA gp38/β-actin ratio set to 100%) and flow cytometry (percentage of gp38(+) RA SF is indicated, *p = 0.03). MFI: Mean fluorescence intensity. (B) Invasive capability of RA SF on matrigel coated transwells expressed as the number of cells per field that migrated through the matrigel at 4 days. (C) SF migration in wound assays expressed as the percentual closure of the wound area 18 h and 24 h after scraping. A representative image is shown (100x). (D) GAG release into the supernatant at 4 and 7 days of RA SF cartilage co-culture. Basal: GAG release by cartilage in the absence of SF. A representative image of siRNA GFP transduced SF attached to cartilage is shown (200x). Results are representative of three independent experiments (NS: not significant). (TIF) [file pone.0099607.s001.tif]

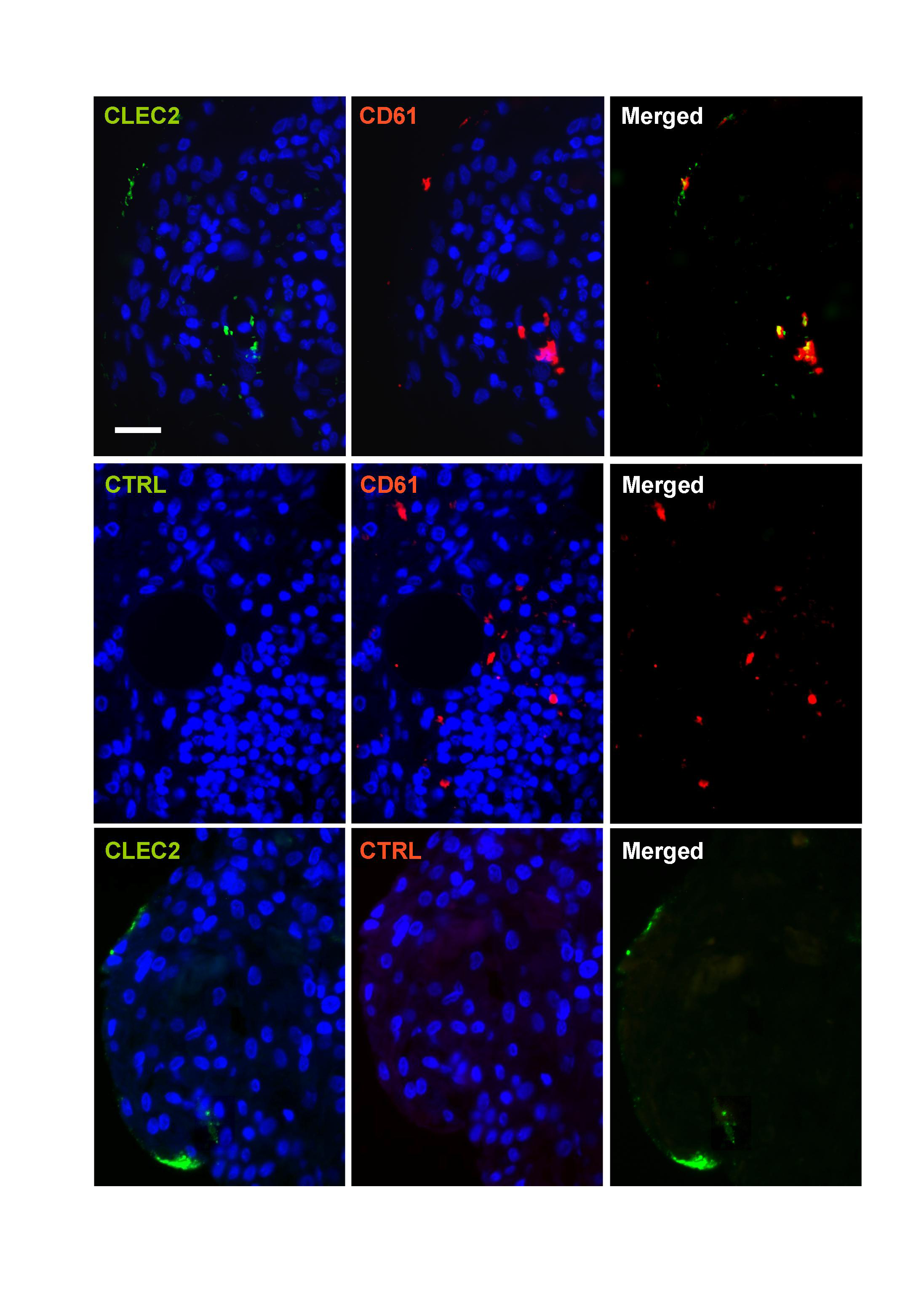

Supplement: Figure S2 — Double CLEC2 and CD61 platelet labeling in RA synovial tissues. Double green (CLEC2) red (CD61) labeling with DAPI counterstaining is shown is shown in upper row as indicated. Controls (CTRL) including only anti-CLEC or only CD61 primary antibody and both secondary antibodies are shown in lower rows as indicated. Bar 20 µm. (TIF) [file pone.0099607.s002.tif]

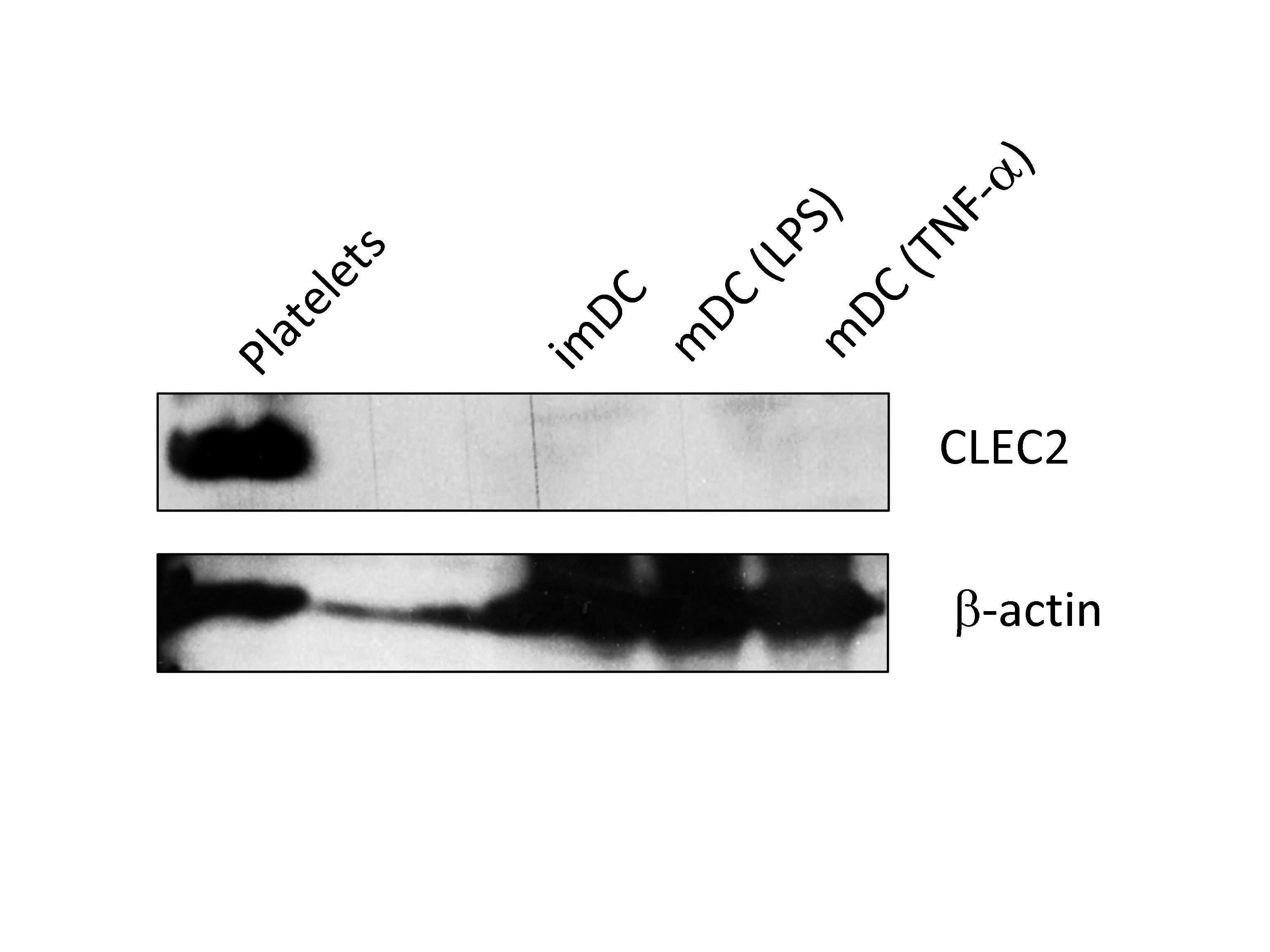

Supplement: Figure S3 — Western blot analysis of CLEC2 expression. Platelets were obtained from healthy donors peripheral blood. Immature DC (imDC) were derived from buffy coat monocytes cultured for 7 days in complete RPMI medium containing GM-CSF (1000 U/ml) and IL-4 (1000 U/ml). For maduration, imDC were treated with LPS (1 µg/ml) or TNF-α (50 ng/ml) for 24 or 72 h respectively. Protein extracts (30 µg) are analyzed by western blotting with anti-CLEC2 polyclonal antibody (R&D Systems) or anti-β-actin mAb (Sigma), developed by peroxidase-conjugated secondary antibodies and ECL system. (TIF) [file pone.0099607.s003.tif]
