# Supplementary material for: Clinicopathological Correlations of Podoplanin (gp38) Expression in Rheumatoid Synovium and Its Potential Contribution to Fibroblast Platelet Crosstalk
Source: PLoS One. 2014 Jun 16;9(6):e99607. doi: 10.1371/journal.pone.0099607 (PMC4059710; doi:10.1371/journal.pone.0099607)
Supplement: Table S1 — Primer sequences used for quantitative real-time PCR analysis. (DOC) [file pone.0099607.s004.doc]

**Table S1**. Primer sequences used for quantitative real-time PCR analysis.

| **Gene Name** | **Forward Primer Sequence** | **Reverse Primer Sequence** | **Amplicon (bp)** |
| --- | --- | --- | --- |
| IL6 | 5'- GTGGCTGCAGGACATGACAA -3' | 5'- TGAGGTGCCCATGCTACATTT -3' | 100 |
| IL8 | 5’- AAGAGCCAGGAAGAAACCACC -3’ | 5’- CTGCAGAAATCAGGAAGGCTG -3’ | 100 |
| gp38 | 5'- CCAGGAACCAGCGAAGACC -3' | 5'- GCGTGGACTGTGCTTTCTGA -3' | 119 |
| CXCL2 | 5’- TCAAACCCAAGTTAGTTCAATCCTGA -3’ | 5’- GCTGACATGTGATATGTCATCACGAA -3’ | 113 |
| CXCL3 | 5'- CGCCCAAACCGAAGTCATAG -3' | 5'- GCTCCCCTTGTTCAGTATCTTTT -3' | 109 |
| CXCL13 | 5'- GAGCCTGTCAAGAGGCAAAG -3' | 5'- CTGGGGATCTTCGAATGCTA -3' | 142 |
| CCL21 | 5'- GTTGCCTCAAGTACAGCCAAA -3' | 5'- AGAACAGGATAGCTGGGATGG -3' | 102 |
| IL7 | 5'- TTCCTCCCCTGATCCTTGTTCT -3' | 5'- CCAATTTCTTTCATGCTGTCCAA -3' | 133 |
| MMP1 | Hs00233958_m1 (TaqMan assay, Applied Biosystems) | | 133 |
| MMP3 | Hs00968305_m1 (TaqMan assay, Applied Biosystems) | | 126 |
| MMP9 | 5'- GGGACGCAGACATCGTCATC -3' | 5'- TCGTCATCGTCGAAATGGGC -3' | 139 |
| β-actin | 5'-GCGCGGCTACAGCTTCAC-3' | 5'-GGCCATCTCTTGCTCGAAGT-3' | 100 |
| β-actin | Human ACTB Endogenous Control (TaqMan assay, Applied Biosystems) | | 171 |

IL6, interleukin 6; IL8, interleukin 8; gp38, glycoprotein 38; CXCL2, chemokine (C-X-C motif) ligand 2; CXCL3, chemokine (C-X-C motif) ligand 3; CXCL13, chemokine (C-X-C motif) ligand 3; CCL21, chemokine (C-C motif) ligand 21 (CCL21); IL7, interleukin 7; Rantes, regulated on activation, normal T cell expressed and secreted; MCP1, monocyte chemotactic protein-1; MMP1, matrix metallopeptidase 1; MMP3, matrix metallopeptidase 3; MMP9, matrix metallopeptidase 9
